# Financial concern reduces child directed speech in a socioeconomically diverse sample

**DOI:** 10.1038/s41598-022-13177-2

**Published:** 2022-06-02

**Authors:** Erin Roby, Rose M. Scott

**Affiliations:** 1 Department of Pediatrics, NYU Grossman School of Medicine, 550 First Ave., New York, NY 10016 USA; 2 Psychological Sciences, University of California Merced, 5200 North Lake Road, Merced, CA 95343 USA

**Keywords:** Psychology, Human behaviour

## Abstract

Socioeconomic status predicts the quantity and nature of child-directed speech that parents produce. However, the mechanisms underlying this relationship remain unclear. This study investigated whether the cognitive load imposed by resource scarcity suppresses parent talk by examining time-dependent variation in child-directed speech in a socioeconomically diverse sample. We predicted that child-directed speech would be lowest at the end of the month when Americans report the greatest financial strain. 166 parents and their 2.5 to 3-year-old children (80 female) participated in a picture-book activity; the number of utterances, word tokens, and word types used by parents were calculated. All three parent language measures were negatively correlated with the date of the month the activity took place, and this relationship did not vary with parental education. These findings suggest that above and beyond individual properties of parents, contextual factors such as financial concerns exert influence on how parents interact with their children.

## Introduction

Research has shown that both the quantity and nature of child-directed speech is associated with numerous aspects of child development including receptive and expressive vocabulary development^1,2^, language processing abilities^3^, the lexical and syntactic diversity of the speech children produce^4^, and children’s school readiness and academic success^5^. Given the broad body of evidence demonstrating the importance of child-directed speech, it is critical to identify potential sources of variability in parent talk.

One factor that has received considerable attention is socioeconomic status (SES). Research suggests that parents of higher SES engage in higher rates of child-directed speech than lower SES parents^6–8^. The striking finding first published by Hart and Risley^6^ showing large SES differences in child-directed speech became widely cited in popular press and academic journals and has since been described as the 30-million-word gap. Although there has been some recent debate regarding the nature of this ‘word gap’^9–11^ several recent meta-analyses have confirmed a relationship between SES and the quantity of language in children’s environment^12,13^. In addition to this quantity difference, recent research has also shown SES differences in the nature of parents’ child-directed speech. For instance, parents with higher SES use more diverse and sophisticated vocabulary and produce more complex sentences and syntactic structures than lower SES parents^2,4,7^.

A question that follows is what might explain the relationship between SES and child-directed speech? One approach that has been taken to answering this question is to focus on properties of the individual parent. For instance, parents with higher levels of education (one dimension of SES) tend to be more knowledgeable about child development^14–16^. Research has in turn shown that parents with less knowledge or skills related to child development engage in less child-directed speech^16–19^ and that the relationship between SES and child-directed speech is mediated by parenting knowledge^16,18^. However, there are several reasons to suspect that this individual-level approach alone cannot fully explain SES differences in child-directed speech. First, interventions focused on enhancing parenting knowledge and skills are not always successful in increasing child-directed speech^20^. Second, mothers with high and low SES view child-directed speech as equally important, but still engage in child-directed speech at different rates^21^. Finally, some research has shown that there is considerable variability in child-directed speech within SES strata^4,7,8,22^, suggesting that there may be external effects on child-directed speech that operate across levels of SES.

An alternative possibility is that SES differences in child-directed speech may be explained in part by broader contextual factors that exert influence on parenting behaviors. In line with this perspective, Conger and colleagues have developed and tested the Family Stress Model to explain how economic pressures affect family functioning and individual adjustment, and consequently, a broad range of child development outcomes^23–25^. More specifically, financial stress is posited to impact aspects of caregiving including warmth, sensitivity, and cognitive stimulation—activities such as reading aloud, play, and other interactions that involve child-directed speech and support children’s language development. For instance, Nievar and colleagues^26^ showed that lower income predicted reduced positive parenting behaviors such as responsiveness to infant vocalizations, which then predicted children’s later cognitive outcomes in the first grade. There is also evidence suggesting links from economic concerns to parenting stress and depression and in turn, child language^26–29^. Moreover, though initially conceptualized to explain family processes among low-SES families, evidence suggests that the Family Stress Model applies to families across varying socioeconomic levels^30,31^. Ponnet^31^ hypothesized that although the nature of financial concerns may differ at different levels of SES, the subjective experience of worrying about finances is likely to impact parenting and family functioning across families from a range of SES backgrounds. Indeed, recent surveys suggest that Americans across the SES continuum are financially stressed. Less than 4 in 10 Americans state that they would be able to pay a surprise $1000 bill from their savings^32^ and of individuals who make over $100,000 a year, over 30% regularly run out of money between paychecks^33^.

The idea that financial concerns impact parents’ capacity to interact with their children complements a growing body of research showing that specific structural sources of stress such as resource scarcity affect adults’ psychological functioning and cognitive processes^34–37^. Mani and colleagues^38^ suggest that when a particular resource is scarce, this taxes cognitive resources and individuals shift their attention to focus on the scarce resource (i.e., tunneling) at the expense of other issues that might require attention. This can lead to counter-productive behaviors such as anxiety, forgetting, attentional neglect, and poor decision making^39–41^. For instance, Shah et al.^40^ conducted a laboratory study in which participants were given either rich (more guesses) or poor (fewer guesses) budgets to play a version of Wheel of Fortune, after which they completed a task measuring cognitive fatigue. Participants with poor budgets demonstrated more cognitive fatigue, suggesting that resource scarcity caused those with poor budgets to increase focus during the game, which in turn resulted in reduced cognitive function on the later task. Additional findings from Shah et al.^36^ speak to how financial concerns influence individuals’ everyday experiences. Through several experiments, Shah and colleagues showed that individuals who were not necessarily poor but had constrained budgets were more likely than higher-income individuals to think about financial concerns when contemplating daily events that involved an economic dimension (e.g., going to the doctor), were more likely to do so unprompted, and found it difficult to suppress thoughts about the cost of common activities (e.g., driving) when instructed not to think about it. Complementary evidence suggests that reducing financial concern can improve cognitive function^39,42^. For instance, Ong et al.^39^ demonstrated that providing debt relief to individuals with chronic debt resulted in improvements in their inhibitory control abilities and reductions in anxiety.

Financial strain is not experienced as a steady state for all families. Instead, it varies over the course of the month due to timing in paydays, distribution of public assistance benefits, and when bills are due. Two recent studies by Carvalho and colleagues showed that in the US, paydays and other income sources (e.g., Disability, Supplemental Security Income, etc.) are distributed 1–2 times per month and are most frequently paid at the beginning of the month^43^. In the state of California, where the present research was conducted, public assistance benefits (e.g., Supplemental Nutrition Assistance Program or SNAP, welfare) are typically paid within the first 3 to 10 days of each month and credit cards and other bills are frequently due at the beginning or middle of each month^44–46^.This timing corresponds to families spending less on groceries and other goods later in the month, when Americans report that they are less financially secure^33,47–49^.

If parent–child interactions are impacted by financial strain, as suggested by the Family Stress Model, then variation in financial strain over the course of the month should be linked to corresponding variation in parent and child behavior. Findings from several recent studies that have used the date of the month of an indicator of financial strain are consistent with this possibility. For instance, one study found that elementary-school children’s disciplinary infractions spiked at the end of the month, and this pattern of findings was most apparent in children whose families receive SNAP benefits, which are distributed early in the month^50^. In addition, reducing financial strain at later points in the month via changes in benefit distribution is associated with reductions in crime and grocery store theft^51^. Finally, the impacts of financial strain on cognitive load have also been linked to payday timing. In a study by Burlacu et al.^52^, priming financial worry among parents impacted their budgeting for immediate household needs as opposed to child learning products such as books, despite the presence of subsidies for the child products. These impacts were most pronounced among those who were furthest from their payday, suggesting that the timing of financial concerns reduced their decision-making capacities.

Building on this body of research, Ellwood-Lowe and colleagues^53^ recently proposed that the cognitive load imposed by resource scarcity could directly suppress parent talk: if parents’ attention is occupied by financial concerns, this leaves less attention to devote to child-directed speech. They tested this hypothesis in two ways. In one study, scarcity was experimentally manipulated in a laboratory setting by prompting caregivers to reflect on times in the prior week when resources were scarce. Parents’ child-directed speech was then examined during a play session and compared to a control group that was not prompted to reflect on scarcity. Not all parents reflected on financial scarcity when prompted, but those who did spoke less to their 3-year-olds than parents who failed to reflect on financial scarcity and parents in the control group. In a second study, Ellwood Lowe et al. examined corpora of day-long in-home recordings to test whether parents’ speech decreased near the end of the month which, as noted previously, is when Americans report experiencing greater financial strain^33,49^. Results showed that parent–child conversational turns tended to be lower in the last week of the month compared to earlier weeks, though this finding only emerged in 1 of the 3 corpora examined. The authors concluded that above and beyond individual characteristics of parents, financial concerns may impact the tendency to engage in child-directed speech.

These findings from Ellwood-Lowe and colleagues^53^ provide initial suggestive evidence for a link between financial concern and parents’ child-directed speech. However, there was limited variation in family SES in both studies. In the first study, the majority of parents were high SES: the median household income was over $150 k per year, and a third of the parents reported a household income over $200 k per year. Although the SES levels in the second study were somewhat lower and more variable than in study 1, the majority of parents were still from middle- to upper-middle class backgrounds. Moreover, the single corpus where the effect of the week of the month was significant happened to have the highest reported family income, and 50% of the mothers in this corpus had a graduate degree (MA or higher). Thus, it remains unclear whether the impact of financial stress on child-directed speech is robust, and whether it operates across SES levels, as the Family Stress Model would predict^25^.

The present study tested the claim that financial concern suppresses child-directed speech. Using two existing corpora of lab-based parent–child interactions from socioeconomically diverse samples (see Table 1 for demographic information), we examined whether parents’ speech to their children varied over the course of the month. As discussed above, Americans tend to spend more money during the first and third week of the month and less in the last week of the month^47^, and report greater financial concern later in the month^33,47–49^. Financial concern is likely to be particularly salient in the state of California, where the present corpora were collected: California is ranked second in the nation for cost of living and is home to 33 million families living paycheck to paycheck^54,55^. Benefits in California are also paid early in the month, and thus financial concern should be greater later in the month^44,45^. We therefore hypothesized that the quantity and diversity of the child-directed speech that parents produced during an interaction with their child would be negatively associated with the day of the month in which the interaction took place. We reasoned that if this pattern emerged in a lab-based interaction where it was salient to parents that their speech to their child was being observed, this would provide particularly strong evidence that financial strain suppresses child-directed speech.

**Table 1:** Demographic characteristics.

	Mean (SD) or *N*
Child age in months—mean (SD); range	31.5 (3.4); 27.1–39.3
Child sex	86 male, 80 female
**Child race**	
White	120
Black/African American	4
Asian	8
Hawaiian or Pacific Islander	1
Native American	1
More than one race	9
Other race	14
NA	9
**Child ethnicity**	
Hispanic or Latino	83
Non-Hispanic or Latino	80
NA	3
**Highest degree completed by either parent**	
Highschool or less	36
Associate’s degree	35
Bachelor’s degree	53
Master’s/PhD/professional degree	42

## Results

Participants were parent–child dyads from two existing corpora in which dyads completed an interactive picture-book activity. Interactions were recorded, transcribed, and coded for three measures of parent language: number of utterances, number of word tokens, and number of word types (see Table 2). We then examined whether these measures varied as a function of the day of the month that the lab visit and parent–child interaction took place (visit day).

**Table 2:** Mean (SD) number of parent utterances, word tokens, and word types in the picture-book interaction, overall and separately by visit week.

	Utterances	Word tokens	Word types
Week 1 (*N* = 38)	131.3 (62.0)	549.6 (248.9)	167.5 (58.8)
Week 2 (*N* = 37)	129.8 (50.2)	545.1 (248.8)	168.2 (53.0)
Week 3 (*N* = 42)	121.4 (59.5)	489.3 (248.7)	155.8 (53.0)
Week 4 (*N* = 49)	101.3 (42.6)	418.8 (199.3)	141.8 (48.2)
Overall (*N* = 166)	119.6 (54.5)	494.7 (239.4)	157.1 (53.7)

Four parents had utterance counts that fell more than 3 standard deviations above the mean for this measure. Excluding these dyads from the analyses of parent utterances did not affect the patterns of significance reported below, so we retained them in the analyses. There were no outliers for word tokens or word types. Six parents spoke Spanish for a portion of the interaction. All of the patterns of significance reported below remain the same if these dyads are excluded. Preliminary analyses revealed no significant effects involving child sex, parent sex, or ethnicity, all *p*s > 0.15. We therefore collapsed across these factors in subsequent analyses.

**Figure 1:**
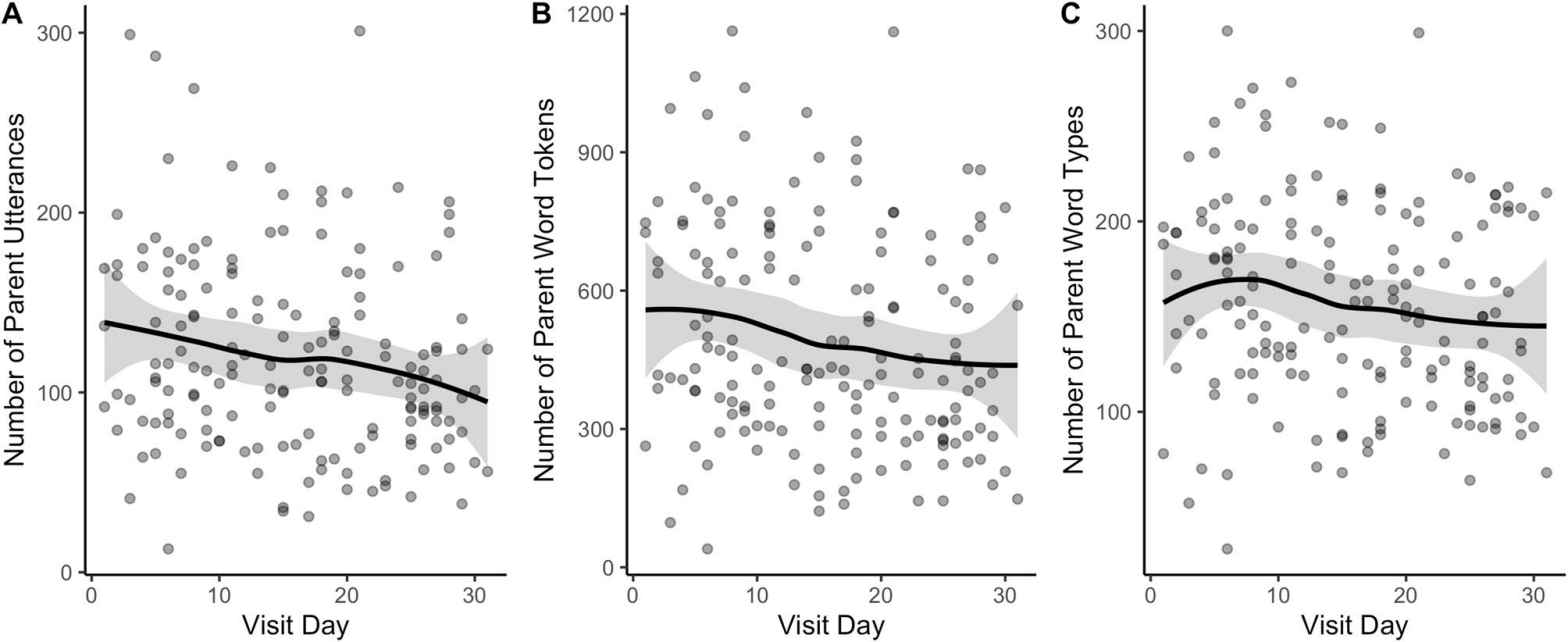
Trend across the day of the month that the visit took place for the number of parent utterances (**A**), word tokens (**B**) and word types (**C**). Dots represent individual participants. The shaded area is the 95% confidence interval around the trend line.

Zero-order bivariate correlations revealed that, as predicted, the day of the month that the parent–child interaction took place was significantly negatively correlated with the number of parent utterances, *r* = − 0.20, *p* = 0.011, word tokens, *r* = − 0.19, *p* = 0.014, and word types, *r* = − 0.16, *p* = 0.04 (see Fig. 1).

To determine whether visit day significantly predicted parent talk after accounting for demographic variables and corpus, a hierarchical multiple regression model was conducted for each parent talk variable. In each model, child age, corpus, and parent education level (three dummy variables for Associate’s, Bachelor’s, and Master’s/PhD, with High school or less as the reference category) were entered at Step 1, and visit day was entered at Step 2. Finally, to test whether the effects of visit day varied across socioeconomic strata, interactions between visit day and parent education level were entered at Step 3. As shown in Table 3, there were no effects of child age or corpus in any of the models. The effect of visit day was significant in all three models, and this effect did not interact significantly with parent education level. Thus, parents who participated later in the month produced less speech and their speech was less diverse, regardless of their level of education.

**Table 3:** Hierarchical multiple regressions predicting parent talk variables from child age, corpus, parent education level, and visit day.

	∆*R*^*2*^	*ß*
**Dependent variable: parent utterances**		
Step 1	0.03	
Child age		0.14
Corpus		− 0.25
Associate’ degree		− 0.07
Bachelor’s degree		− 0.01
Master’s/PhD		0.01
Step 2	0.04*	
Visit day		− 0.20*
Step 3	0.01	
Visit day × Associate’s degree		− 0.04
Visit day × Bachelor’s degree		− 0.22
Visit day × Master’s/PhD		− 0.12
**Dependent variable: parent word tokens**		
Step 1	0.02	
Child age		0.17
Corpus		− 0.16
Associate’ degree		− 0.04
Bachelor’s degree		0.09
Master’s/PhD		0.09
Step 2	0.04*	
Visit day		− 0.20*
Step 3	0.02	
Visit day × Associate’s degree		− 0.09
Visit day × Bachelor’s degree		− 0.37
Visit day × Master’s/PhD		− 0.26
**Dependent variable: parent word types**		
Step 1	0.03	
Child age		0.18
Corpus		− 14
Associate’ degree		0.05
Bachelor’s degree		0.14
Master’s/PhD		0.17^^^
Step 2	0.03*	
Visit day		− 0.18*
Step 3	0.02	
Visit day × Associate’s degree		− 0.02
Visit day × Bachelor’s degree		− 0.37
Visit day × Master’s/PhD		− 0.25

^^^*p* < 0.10; *p < 0.05; *ß* = standardized regression coefficient.

**Figure 2:**
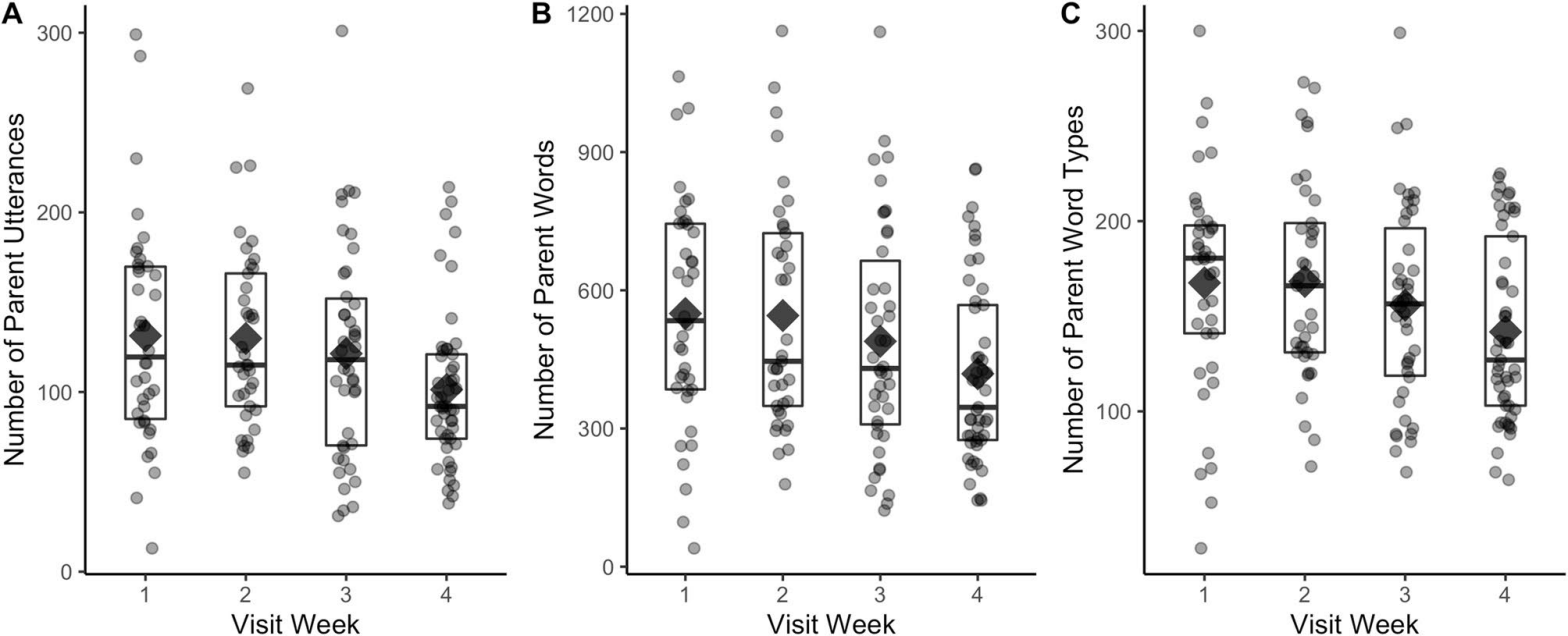
Number of parent utterances (**A**) word tokens (**B**) and word types (**C**), separately by visit week. Dots represent individual participants, diamonds indicate the mean, boxes represent the interquartile range, and the horizontal line in each box indicates the median.

To facilitate comparisons with Ellwood-Lowe et al.^53^, we compared parent talk during the last week of the month (days 22–31) to the rest the month (days 1–21; see Fig. 2). For each parent talk variable, we conducted an analysis of variance (ANOVA) with visit week (week 4 vs weeks 1–3) and parent education (High school or less, Associate’s, Bachelor’s, Master’s/PhD) as between-subjects factors. These models revealed a significant effect of visit week on the number of parent utterances, *F*(1, 158) = 5.82, *p* = 0.017, *η*^2^ = 0.04, and the number of parent word tokens, *F*(1, 158) = 4.23, *p* = 0.041, *η*^2^ = 0.03, and a marginal effect of visit week on the number of parent word types, *F*(1, 158) = 3.66, *p* = 0.058, *η*^2^ = 0.03. There were no significant effects of parent education (utterances: *F*(3, 158) = 0.17, *p* = 0.92; tokens: *F*(3, 158) = 0.33, *p* = 0.81; types: *F*(3, 158) = 0.24, *p* = 0.87) and no significant interactions of visit week and parent education (utterances: *F*(3, 158) = 0.36, *p* = 0.78; tokens: *F*(3, 158) = 0.96, *p* = 0.41; types: *F*(3, 158) = 1.45, *p* = 0.23).

Because time of the month was not a factor of interest in the original studies from which these data were derived, the timing of study visits was not controlled: appointments were scheduled on the day the parents chose. This raises the possibility that visit day was confounded with other participant characteristics that could impact parent talk. A final set of analyses indicated that visit day did not vary as a function of child sex, parent sex, ethnicity, or corpus, all *F*s < 1, all *p*s > 0.76. There was a marginal effect of parent education on visit day, *F*(3, 162) = 2.54, *p* = 0.058, *η*^2^ = 0.05. Examination of this marginal effect revealed that the visit day of parents in the High school or less group was somewhat earlier in the month (*M* = 12.5) than the visit day of parents in the other three education groups (Associate’s *M* = 17.6; Bachelor’s *M* = 16.7; Master’s/PhD *M* = 15.8). Although the reason for this sampling difference is unclear, the direction of this effect means that the lower levels of parent speech observed on later visit days were not due to oversampling low-SES parents later in the month, as these parents tended to participate earlier in the month. More critically, all of the significant relationships between parent talk and visit day held after controlling for parent education, and no interactions emerged between visit day and parent education in any of our analyses. Together, these analyses suggest that the relationship between visit day and parent talk was not driven by any of the participant variables that were available in the data set.

## Discussion

Considerable evidence suggests that parents’ engagement in child-directed speech is related to a number of important aspects of child development, and in particular, language development^1,2,22^. It is therefore critical to consider potential sources of variability in parent talk. In the current study we examined one possible source of variation that has been hypothesized to exert influence on parents’ use of child-directed speech: financial concern^28,53^. We used existing corpora of lab-based parent–child interactions and examined whether parents’ speech to their children varied over the course of the month. We predicted that parents would talk less near the end of the month when financial concerns were likely to be greatest^33,47–49^. Consistent with this hypothesis, results showed that the number of utterances, word tokens, and word types that parents produced were negatively correlated with the date of the month when they participated in the parent–child interaction. These associations remained after accounting for child age, corpus, and parental education. Finally, as one would expect if financial strain were highest at the end of the month, parents who participated in the final week of the month produced significantly fewer utterances and word tokens and marginally fewer word types than parents who participated earlier in the month.

These findings provide the first evidence that the time during the month that a parent–child interaction occurs relates to parents’ use of child-directed speech in a socioeconomically diverse sample. Parents in the current study varied considerably in their level of education (see Table 1) and they were drawn from a region where families tend to have lower socioeconomic status than other parts of the state or country. The current findings therefore replicate those reported by Ellwood-Lowe and colleagues^53^, who tested primarily middle- to upper-middle class families, and extend them to a more socioeconomically diverse sample. The fact that time-dependent differences in child-directed speech emerged in a more socioeconomically diverse sample suggests that the impact of financial concern on child-directed speech is robust and operates across SES levels. These findings suggest that above and beyond individual properties of parents, broader contextual factors such as financial concerns exert influence on the way parents interact with their children.

Our findings are consistent with the Family Stress Model, which posits that economic hardships, such as low income or high debt-to-asset ratio, increase economic pressures, which negatively affect family functioning and parent adjustment, and consequently, child development outcomes^23–25^, including language^27–29^. Although we did not directly measure financial strain, we hypothesize that in the current study, parents participating at days later in the month were more occupied with financial concerns, which affected the speech they used in interactions with their child. If this is the case, then these results suggest that economic strain plays a role in parents’ capacity to interact with their children. Moreover, our analyses revealed no effect of education on parents’ child-directed speech, suggesting that families from high- and low-SES backgrounds were similarly influenced by the date the interaction took place, as well as the potential corresponding economic pressure. Although families from low- and high-SES backgrounds likely experience financial strain very differently from one another, the current findings suggest that financial strain can nevertheless have impacts on parental language across SES levels. Our results therefore add to the growing body of findings suggesting that these processes are not specific to families living in poverty^31^, and that the Family Stress Model applies to families experiencing financial concerns across socioeconomic strata.

The current findings also extend prior work by showing that parents’ child-directed speech was lower at later points in the month in a context where it was particularly salient that they were being observed. Parents were given instructions on how to interact with their child and then were left in a room where video recording devices were clearly visible. The fact that parental talk varied across the month despite these cues and despite the potential urge to engage in high levels of child-directed speech due to social desirability bias is well-aligned with the hypotheses set forth by Mani and colleagues regarding scarcity^34–37^. In particular, these findings complement laboratory and field research showing how financial concern operates in everyday life and that these cognitions can arise spontaneously and are difficult to suppress^36^. In the case of the current study, the influence of financial concerns surfaced even in the face of cues that might otherwise enhance parental talk.

Our finding that parents engage in less child-directed speech at later points in the month is consistent with those reported by Ellwood-Lowe and colleagues^53^, who examined day-long recordings collected in the home. However, this latter finding was only significant in one of the three corpora examined. This pattern of results raises the possibility that the influence of financial concern on parent speech might be greater in laboratory settings than at home. Contrary to our suggestion that being observed might have enhanced parent talk, one might instead argue that being in a laboratory could enhance effects of financial concern because parents experiencing more stress might be less able to “perform” under scrutiny. If so, then the impact of financial concern on child-directed speech might be less evident in home environments. Although possible, prior research examining the speech used in parent–child interactions has revealed converging findings across laboratory and home assessments^12,56–60^^.^ In particular, recent work examining the association between SES and child-directed speech found that location of the assessment (laboratory vs. home) did not significantly moderate this relationship^12^. This converging evidence across methods suggests that lab-based measures of parent language, such as the picture-book task used in this study, tap into consistent patterns of behavior that are reflective of children’s everyday experiences. We therefore think it likely that our findings reflect a more general impact of financial concern on parents’ child-directed speech that would also emerge in the home setting. Nevertheless, it would be worthwhile to replicate the present findings in naturalistic settings to clarify in which contexts financial concern impacts parents’ child-directed speech.

A second question is whether the current findings would generalize to other participant samples. The current findings and those reported elsewhere suggest that in the US, the effect of financial concern on parent–child interactions is not specific to lower SES families. However, this effect might differ for very low SES families, such as those living well below the poverty line. Notably, some racial and ethnic minority groups experience higher levels of stress, earn lower wages, and are more likely to live in poverty^61–63^. This also raises the question of whether our results would emerge across racial and ethnic groups in the US. In the present study, approximately half of the participants were Hispanic or Latino, but we found no effects of ethnicity. However, we did not have information regarding parents’ immigration status, country of origin, or levels of acculturation, which could affect parents’ child-directed speech^64–66^. Similarly, we only examined the quantity of parent talk. Some research suggests that the style and content of parents’ language varies with ethnicity^67^. It is therefore possible that other aspects of parental talk might vary by day of the month and ethnic background.

In terms of the broader global population outside the United States, prior research has shown that the timing of paydays and harvest schedules that are likely to produce financial stress in non-Western cultures correspond to changes in performance on cognitive tasks^35,39,68,69^. These findings could indicate that scarcity impacts cognitive function similarly across cultures, but it remains an open question whether this would also impact parent speech and in turn, child language and other developmental outcomes. In studies within the United States, there is strong evidence showing associations between SES, children’s language environment, and children’s language development. In contrast, in other parts of the world such as in Southern Mexico and Bolivia, children are exposed to less child-directed speech, and despite this, achieve language learning milestones on a typical trajectory consistent with children in the United States^70–72^. Given the infrequency of child-directed speech, it is possible that financial concerns may not impact parents’ speech or child development in these communities. However, scarcity might still impact other aspects of cognitive functioning that have implications for parenting (e.g., attention, organization, planning, etc.). Our results cannot speak to these possibilities. There is thus a need for further work examining whether and how resource scarcity relates to child-directed speech, and caregiving behaviors more broadly, in other socioeconomic and cultural contexts.

One key limitation in the current study is that we did not collect any measures of household income or parent occupation, which have the potential to impact parents’ child-directed speech. While some have argued that of SES indicators, parental education is most predictive of parents’ speech^7^, others suggest that different SES indicators represent distinct constructs with unique linkages to parent outcomes and hence they should not be used interchangeably^73^. Therefore, future research examining how financial concern relates to parents’ speech should test whether additional measures of SES interact with parental talk.

A second limitation is that we lacked a direct measure of finances or financial concern. In the current study, we posited that it was financial strain specifically that contributed to time-dependent differences in parents’ child directed speech. None of the participant characteristics in our data set varied with visit day except for a marginal difference in parent education, and our results held after controlling for that variable. It is possible that other factors that vary with time (e.g., work- or school-related deadlines, health issues, etc.) could have impacted parental language. However, Americans report that finances are their greatest source of stress^33^, and this source of stress seems the most likely to vary with the time of the month in which parent–child interactions occurred. We therefore conclude that financial concerns are the most probable factor that contributed to differences in parental language over the course of the month. Still, the effect sizes in the present study were modest, which could reflect our indirect measure of financial concern. Future research on this topic should include direct measures of perceived financial strain and family finances such as when parents were paid, whether families were receiving public assistance, or carrying debt. In addition to providing direct evidence for the impact of financial strain on parent talk, this might yield larger associations between financial concern and child-directed speech. However, collection of this information must be done with caution, as prior work has shown that priming parents to think and talk about financial concern results in reductions in their child-directed speech^53^.

In conclusion, our study indicates that parents talk less to their children at later points in the month, when financial concern is likely to be greatest. Our findings thus suggest that efforts to improve child language outcomes might benefit from focusing on broader contextual factors, such as reducing parents’ financial strain, rather than solely intervening on individual-level factors (i.e. parenting knowledge). The present study contributes to a growing body of evidence that financial strain impacts a range of parent and child behaviors^50–53^. Together, these findings suggest that reducing financial strain could have broad positive impacts on families, above and beyond enhancing child-directed speech and child language outcomes.

## Method

### Participants

This study drew on two existing corpora of parent–child interactions that were part of larger lab-based studies on children’s social-cognitive development^74,75^. These corpora were selected because (a) the same lab-based parent–child activity was used and (b) both samples were socioeconomically diverse. The dyads in the two corpora were recruited from birth records provided by the California Department of Public Health and a database of parents living in Merced County, California who had previously expressed interest in participating in research studies with their children. Parents in the latter group were largely recruited at a variety of community events, many of which focused on providing resources and support for low-SES families. These procedures have been used in several prior studies to recruit socioeconomically and ethnically diverse samples^67,76–78^. Dyads were recruited for a single lab visit and appointments were scheduled at parents’ convenience. Timing of study visits over the course of the month was not controlled because this was not a factor of interest in the original studies. Parents were not paid for their participation; instead, all children received a small gift of approximately $5 value for participating.

Dyads who met the following criteria were included in the present study: (a) they completed the relevant parent–child interaction, (b) children had no known speech, language, or developmental delays, and (c) children were exposed to English at least 50% of the time. The final data set consisted of 166 parent–child dyads. Demographic information for this sample is provided in Table 1. The majority of children completed the tasks with their mother (*N* = 132); the remainder completed the tasks with their father (*N* = 34). On average children were exposed to English 95% of the time (*SD* = 10.29%, range: 50–100%). Over 95% of the sample heard English at least 75% of the time (the patterns of significant in the Results section remain the same if the percentage of English exposure is controlled for).

Information on race, ethnicity, and socioeconomic status was obtained via parental report. Socioeconomic status can be accessed via a variety of indicators, including parents’ education, occupation, and income^73,79,80^. These existing corpora did not include measures of family income or occupation, but did include a measure of parental education. As can be seen in Table 1, the highest degree completed by either parent was diverse, ranging from completing high school or less to possessing professional or advanced degrees, with a relatively even distribution across the education categories. Although income was not measured, the participants were drawn from a region where families are generally relatively low in socioeconomic status. The county where these participants were recruited has a poverty rate of 23% for children 18 years of age and under^81^, which is higher than both the state (16%) and national level (17%). The median household income ($53,672) is also lower than those reported at the state ($75, 235) and national level ($62,843)^81^.

### Procedure

Dyads participated in a single lab visit lasting less than 45 min; children completed one or more social cognition tasks and then dyads completed one or more interactive activities. The present study focused on a picture-book activity that was common across the two corpora. Dyads sat together in a chair or on the floor and viewed a wordless picture book that was adapted from prior research on parent–child mental-state talk^82^. The book contained color photos of children and adults engaged in a variety of activities (e.g., feeding ducks, playing with blocks). The pages did not contain any words. Parents were instructed to go through the book with their child as they would at home. Parents were allowed to use whichever language(s) they felt most comfortable with. The experimenter then left the dyad alone to view the book; the interaction was video recorded.

The length of the book and the duration of the activity varied across corpora. In one corpus^75^ the book was 23 pages and parents were encouraged to take as much time as they wanted; in the other^74^, the book was 10 pages and parents were told that the experimenter would come back after 10 min to conclude the activity.

For each interaction, parent language was transcribed verbatim by trained research assistants who used a formal protocol based on prior language research to guide their work^59,83^. To ensure accurate identification of words and utterances, all transcribers were trained to criterion before beginning the transcription process (i.e. at least 85% agreement with a gold standard coder on at least two training transcriptions). Utterances were identified by grammatical closure, intonation, or pauses. Non-verbal sounds (e.g., gasps, sighs, groans, laughs, non-word sound effects, etc.) were not transcribed. Words that referred to sounds such as animal sounds (e.g., “woof”, “baa”, “moo”) were included in the transcription. Six parents spoke in Spanish for a portion of the task. For these parents, the interaction was first transcribed verbatim by a native Spanish–English bilingual transcriber, and then the Spanish words were translated into English. We then used the CLAN program^84^ to calculate three measures for each parent: number of utterances, number of word tokens, and number of word types. Descriptive information for these variables appears in Table 2.

We also calculated the total duration of each interaction. This was defined as the time between the onset of the first utterance about the book to the offset of the final utterance about the book. The average duration of the interactions was approximately 6 min (*M* = 6.11, *SD* = 2.62, range = 1.93–13.97 min). Despite differences in administration of the task, the duration of the interaction did not vary significantly across the two corpora (corpus 1: *M* = 6.25 min, *SD* = 2.52; corpus 2: *M* = 5.84 min, *SD* = 2.80), *t*(164) = 0.96, *p* = 0.34. Only 10% (*n* = 17) of the interactions had durations longer than 10 min.

### Ethical approval

The right of the subjects who participated in our study were protected. The research was conducted in accordance with the Common Rule guidelines established by the US Office of Human Research Protections. The University of California Merced Institutional Review Board approved all procedures (UCM-10-0027, UCM14-0039). All parents provided written informed consent prior to participation.

## Supplementary Information

Supplementary Information.

## Data Availability

All data generated or analyzed during this study are included in this published article (and its Supplementary Information files).
